# Small RNA data set that includes tRNA-derived fragments from Jurkat cells treated with camptothecin

**DOI:** 10.1016/j.dib.2018.01.050

**Published:** 2018-02-03

**Authors:** Mehmet Ilyas Cosacak, Ipek Erdogan, Ayten Nalbant, Bunyamin Akgul

**Affiliations:** a Izmir Institute of Technology, Department of Molecular Biology and Genetics, Gulbahcekoyu, Urla, 35430 Izmir, Turkey; b German Center for Neurodegenerative Diseases Dresden, DFG-Center for Regerenative Therapies Dresden, Cluster of Excellence, TU Dresden FetscherstraBe 105, 01307 Dresden, Germany

## Abstract

In this article, we report a small RNA data set obtained from human T cell acute leukemia Jurkat cells, which were treated with the universal apoptotic agent camptothecin. Based on the Annexin-V labeling pattern, we sorted two Jurkat subpopulations in treated cells: one that is sensitive to the drug and the other being relatively more resistant. We report new original data that include the frequency of tRNA-derived fragments (tRF) in drug-sensitive and resistant cells. We also present partially analyzed data to show the origin of reads on tRNAs as well as the borders of the fragments. We believe that this data can benefit the science community working in the field of tRF and/or apoptosis.

**Specifications Table**

**Table:** 

Subject area	Biology
More specific subject area	Molecular Genetics
Type of data	Figures and Excel file
How data was acquired	Deep sequencing of total RNAs
Data format	Analyzed data
Experimental factors	Drug treatment and cell sorting
Experimental features	Total RNAs isolated from sorted cells were subjected to deep-sequencing using Illumina platform
Data source location	Izmir, Turkey
Data accessibility	Raw data available at GEO (Accession number GSE35442)

**Value of the data**

• These data can be used to study drug-induced fragmentation pattern of tRNAs.
• The abundance of tRNA-related fragments in drug-sensitive and resistant cells can be used to study potential tRF-mediated regulation of apoptosis.
• These data are also useful for the researchers interested in the discovery of novel tRNA-related apoptotic markers.

## 1 Data

**Fig. 1:**
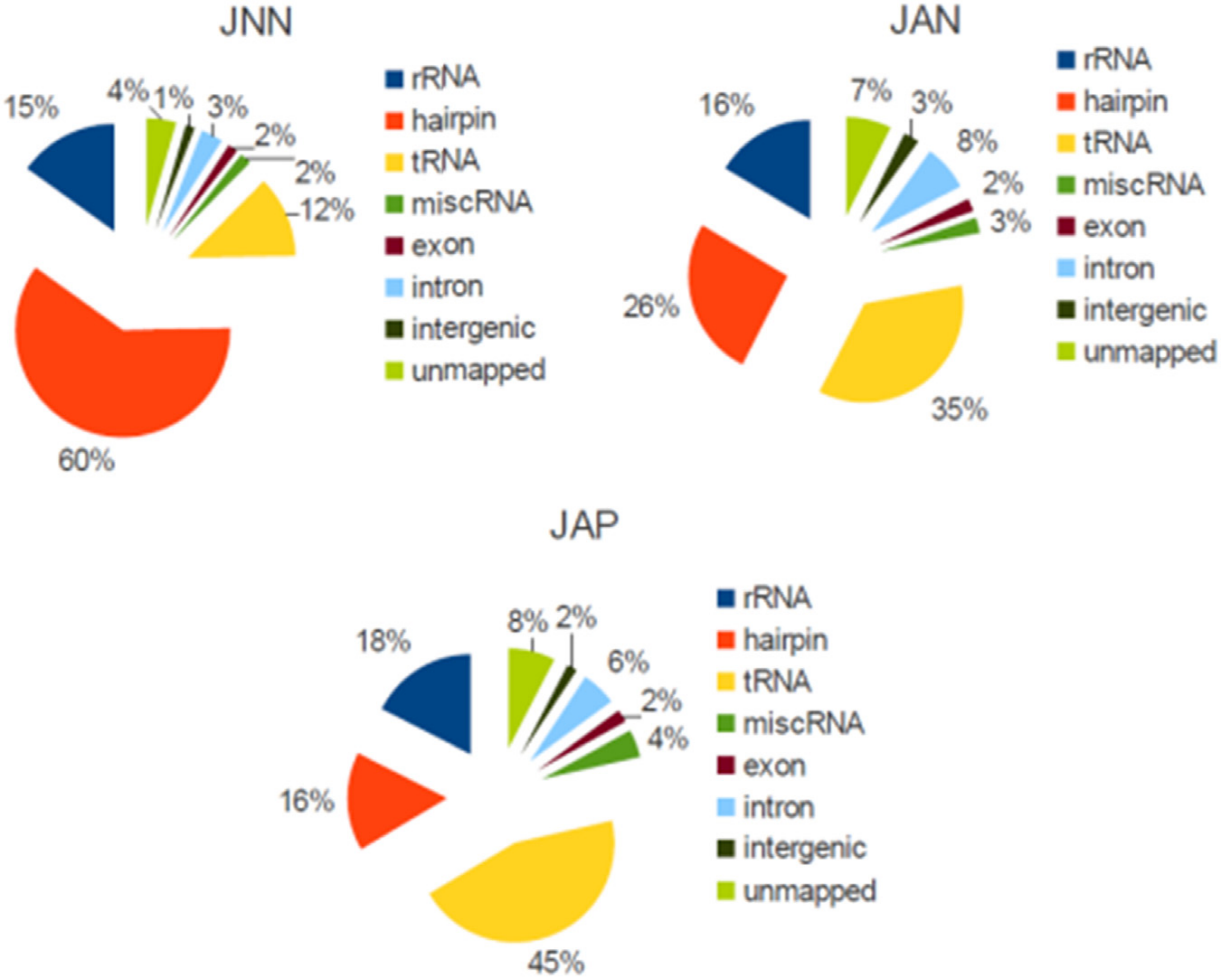
The percentage of different small RNA populations in the drug-free, control cells (JNN), camptothecin-treated and Annexin V-positive cells (JAP) and camptothecin-treated and Annexin V-negative cells (JAN).

**Fig. 2:**
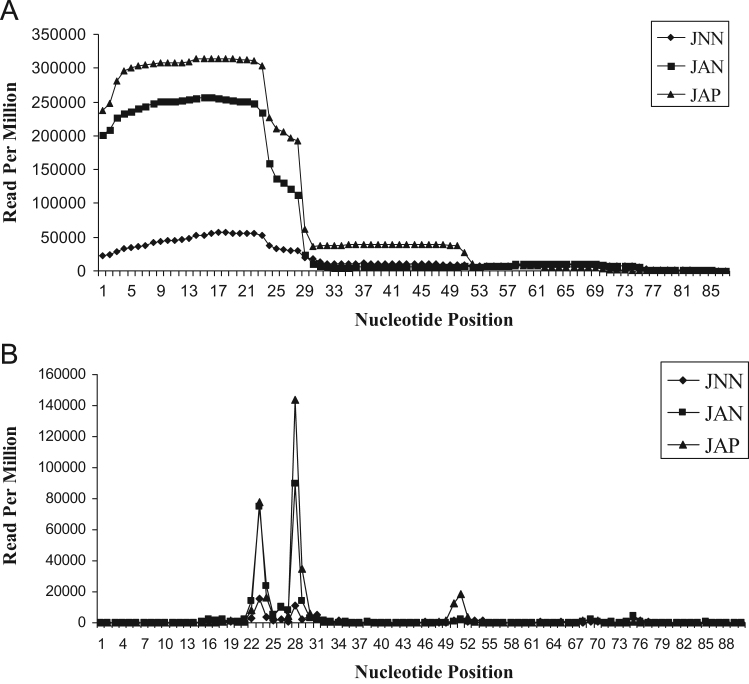
Camptothecin-induced tRNA fragmentation in Jurkat cells. (A) Cloning frequency of each nucleotide on a representative tRNA. (B) The position of 3′ terminus of tRNA-derived fragments relative to their 5′ terminus.

We report three pieces of data. The data in Fig. 1 show the percentage of small RNA reads in each sub-population. Panel A in Fig. 2 is a graph of tRNA-derived fragments superimposed onto a representative tRNA. Panel B in Fig. 2 displays the potential 3′ cleavage site of tRNA-derived fragments on a representative RNA. Expression levels of tRNA-derived fragments are listed in a supplementary Microsoft Excel Table (Supplementary Table 1).

## 2 Experimental design, materials and methods

Jurkat cells were treated with camptothecin (8 uM for 4 h) or incubated in media (control). A subpopulation of cells remained resistant to apoptosis despite the treatment with 128-fold drug concentration. Each population was sorted using Annexin-V-conjugated magnetic beads, resulting in three types of cells: (1) untreated control cells (JNN, Jurkat negative negative); (2) drug-treated but Annexin-V-negative cells (JAN, Jurkat apoptosis negative); and (3) drug-treated, apoptosis, Annexin-V-positive cells (JAP). Total RNAs isolated from each sample type were subjected to deep-sequencing and reads were blasted to the human genome to identify the read frequency of tRNA-derived sequences [1].
